# The influence of Neanderthal alleles on cytotoxic response

**DOI:** 10.7717/peerj.5691

**Published:** 2018-10-23

**Authors:** Farida S. Akhtari, Tammy M. Havener, Masahide Fukudo, John R. Jack, Howard L. McLeod, Tim Wiltshire, Alison A. Motsinger-Reif

**Affiliations:** 1 Department of Biological Sciences, North Carolina State University, Raleigh, NC, United States of America; 2 Bioinformatics Research Center, North Carolina State University, Raleigh, NC, United States of America; 3 Pharmacotherapy and Experimental Therapeutics, University of North Carolina at Chapel Hill, Chapel Hill, NC, United States of America; 4 Department of Pharmacy, Kyoto University, Kyoto, Japan; 5 Department of Statistics, North Carolina State University, Raleigh, NC, United States of America; 6 The DeBartolo Family Personalized Medicine Institute, Moffitt Cancer Center, Tampa, FL, United States of America; 7 Center for Pharmacogenomics and Individualized Therapy, University of North Carolina at Chapel Hill, Chapel Hill, NC, United States of America

**Keywords:** Neanderthal alleles, Cytotoxic response, Pharmacogenomics, Toxicogenomics, Dose responses, Neanderthal ancestry, Lymphoblastoid cell lines, Anti-cancer drugs, Environmental chemicals, Single nucleotide polymorphisms

## Abstract

Various studies have shown that people of Eurasian origin contain traces of DNA inherited from interbreeding with Neanderthals. Recent studies have demonstrated that these Neanderthal variants influence a range of clinically important traits and diseases. Thus, understanding the genetic factors responsible for the variability in individual response to drug or chemical exposure is a key goal of pharmacogenomics and toxicogenomics, as dose responses are clinically and epidemiologically important traits. It is well established that ethnic and racial differences are important in dose response traits, but to our knowledge the influence of Neanderthal ancestry on response to xenobiotics is unknown. Towards this aim, we examined if Neanderthal ancestry plays a role in cytotoxic response to anti-cancer drugs and toxic environmental chemicals. We identified common Neanderthal variants in lymphoblastoid cell lines (LCLs) derived from the globally diverse 1000 Genomes Project and Caucasian cell lines from the Children’s Hospital of Oakland Research Institute. We analyzed the effects of these Neanderthal alleles on cytotoxic response to 29 anti-cancer drugs and 179 environmental chemicals at varying concentrations using genome-wide data. We identified and replicated single nucleotide polymorphisms (SNPs) from these association results, including a SNP in the *SNORD-113* cluster. Our results also show that the Neanderthal alleles cumulatively lead to increased sensitivity to both the anti-cancer drugs and the environmental chemicals. Our results demonstrate the influence of Neanderthal ancestry-informative markers on cytotoxic response. These results could be important in identifying biomarkers for personalized medicine or in dissecting the underlying etiology of dose response traits.

## Introduction

Research has revealed that anatomically modern humans and archaic hominins, such as Neanderthals and Denisovans, coexisted and interbred (Green et al., 2010; Higham et al., 2014; Prüfer et al., 2014; Sankararaman et al., 2014). The Neanderthal introgression has led to modern Eurasian genomes containing ∼1 to 4% of DNA inherited from Neanderthals (Sankararaman et al., 2012; Wall et al., 2013; Vernot & Akey, 2014). Recent studies have shown that Neanderthal haplotypes and alleles are under strong selective pressures, with regions undergoing both positive and negative selection (Abi-Rached et al., 2011; Vernot & Akey, 2014; Sankararaman et al., 2014). Importantly, these variants have been shown to be strongly associated with a number of clinically important diseases and phenotypes, such as hypercoagulation, depression, actinic keratosis, and urinary tract disorders among others (Simonti et al., 2016). These associations were discovered and replicated in electronic medical records data. Several of the single nucleotide polymorphisms (SNPs) in the Neanderthal PheWAS catalog are listed as being associated with drug response phenotypes such as ‘Adverse drug events and allergies’, ‘Adverse effects of sedatives or other central nervous system depressants and anesthetics’, ‘Antineoplastic and immunosuppressive drugs causing adverse effects’ and ‘Allergic/adverse effects of penicillin’. This inspired us to investigate the effect of these Neanderthal alleles on drug or chemical response using the Lymphoblastoid Cell Lines (LCL) model.

Drug and chemical dose response are clinically important traits that are influenced by genetic factors. Variability in response to a drug or chemical is observed across individuals and populations, and it has been demonstrated that genetic variation is responsible for much of this variation. Understanding the genetic factors responsible for the variability in individual response to a drug or a chemical is what defines pharmacogenomics and toxicogenomics. The understanding of this variability in response is required to tailor medications to an individual or a population, thus leading to optimized clinical treatments (Ritchie, 2012). There are a number of high impact translational examples in this important area of gene mapping (Ritchie, 2012; McCarthy, McLeod & Ginsburg, 2013; Motsinger-Reif et al., 2013).

While many of the studies in pharmacogenomics and toxicogenomics focus on humans, a large proportion of studies rely on model systems. In particular, model systems allow for precise measurements of response, including mechanistic changes, but also very few studies in humans are designed to specifically measure xenobiotic responses that are known to induce a toxicity phenotype. Mostly, these data have been collected from retrospective and observational studies. Lymphoblastoid Cell Lines (LCLs) provide a convenient model for xenobiotic testing with their associated accumulated genomic data. However, they still have limitations due to their inability to provide response data where metabolism of compounds is an important part of the response, or where whole organism functions, such as immune response, are important.

In this study, we use LCLs as a model of cytotoxic response. The use of LCLs over clinical trials for pharmacogenomics studies has several advantages. LCLs from the CEPH pedigrees (Dausset et al., 1990), the International Hapmap Project (International HapMap Consortium, 2003), The 1000 Genomes Project ( The 1000 Genomes Project Consortium, 2015) and so on are publicly available resources with extensive genomic data. As opposed to traditional pharmacogenomic studies conducted via clinical trials, using the LCL model is more scalable, efficient and cost-effective. The LCL model is conducive to robotic automation for high-throughput assays. Also, there are fewer limitations with LCL models with respect to sample size and confounding effects from treatment variability (Wheeler & Dolan, 2012). The LCL model has been indicated to possess numerous successes (Huang et al., 2008; Stark et al., 2010; Watson et al., 2011; Brown et al., 2012a) and several of the results from these studies have been identified as clinically relevant and they include the association of SNPs in the MGMT gene with temozolomide response (Brown et al., 2012a), the identification of QTLs associated with dose response for 29 FDA-approved drugs (Peters et al., 2011) and the identification and evaluation of SNPs as predictors for platinum response in head and neck cancer patients (Ziliak et al., 2011).

Research has demonstrated that ethnicity and race influence the dose response phenotype, both in clinical samples and in the LCL model of drug response (Ortega & Meyers, 2014). Recently, we have shown strong differences in drug response in the LCL model for both pharmaceuticals and environmental chemicals. Differences in dose response phenotypes were observed between Hispanic and Caucasian populations for 28 chemotherapy drugs (Jack et al., 2015) and significant variation in EC_10_ was observed across the globally diverse populations from the 1000 Genomes Project (1000G) for 179 environmental chemicals (Abdo et al., 2015).

However, the influence of Neanderthal ancestry on dose response has not been interrogated. In this study, we examine if introgressed Neanderthal DNA influences the dose response phenotype in the LCL model. We assessed the effects of genome-wide Neanderthal alleles on cytotoxic response in 520 LCLs derived from unrelated Caucasian individuals from the Children’s Hospital of Oakland Research Institute (CHORI) to 29 anti-cancer drugs, each assayed at six different concentrations (Brown et al., 2014) and in 1,086 LCLs from the 1000 Genomes (1000G) Project to 179 environmental chemicals, each assayed at eight different concentrations (Abdo et al., 2015). We first identified common Neanderthal variants that influence dose response in LCLs derived from Caucasian cell lines from CHORI. We then replicated this result in LCLs derived from the globally diverse 1000G Project. We also ascertain the cumulative effect of Neanderthal alleles on dose response using burden tests, again using the LCLs derived from Caucasian cell lines from CHORI as our discovery dataset and the LCLs from the 1000G Project as our replication dataset.

## Materials and Methods

To identify Neanderthal derived SNPs in the genome-wide dose response data, we used the Neanderthal SNPs listed in the Neanderthal PheWAS catalog (Simonti et al., 2016) which listed 1328 unique Neanderthal derived SNPs. These Neanderthal SNPs were identified by comparing the sequences from the 1000 Genomes Project ( The 1000 Genomes Project Consortium, 2015) with the Altai Neanderthal genome (Prüfer et al., 2014; Vernot & Akey, 2014). The Neanderthal PheWAS catalog provides the association results of 1328 Neanderthal SNPs with 1152 electronic health record (EHR) –derived phenotypes (Simonti et al., 2016).

We used the genome-wide association mapping results and dose response profile data from two previous studies. The first was from a study by Brown et al. (2014), which we used as our discovery dataset. This study provided data from 520 Caucasian cell lines in response to 29 chemotherapy drugs, each assayed at six different concentrations, with 2.1 million SNPs used in the association analysis. Details of the drugs and concentrations used in this study are listed in Table S1. Details of the methods used to assess drug response can be found in Brown et al. (2014). Potential batch effects were controlled by both the experimental design and by our quality control procedures. Briefly, cell lines were assayed in random order with each cell line being seeded on two 384 well plates with 4,000 cells per well. Each plate also included a control for background fluorescence signal, 10% dimethyl sulfoxide (DMSO) and another control for drug vehicle, water or DMSO at 0.01, 0.1, 1 and 2 percent. Each cell line was incubated with each of the 29 anti-cancer drugs at each of the six concentrations for 72 h, dyed with Alamar Blue (BioSource International) and incubated for another 24 h. At this point, LCL viability measurements were taken using an Infinite F200 microplate reader with Connect Stacker (Tecan Group Ltd, Männedorf, Switzerland) and iControl software (Version 1.6), which measures fluorescence intensity in raw fluorescence units (RFUs) at EX535nm and EM595nm. All individuals in this study were genotyped using HumanHap300 bead chip or HumanQuad610 bead chip platforms and these markers were used to impute 2.5 million SNPs from HapMap Release 22, using the Caucasian CEPH reference population. Genotypic quality control on these 2.5 million SNPs resulted in ∼2.1 million SNPs per drug for association analysis. The second set of dose response data was obtained from a study by Abdo et al. (2015), which we used as our replication dataset. This study provided data from 1,086 LCLs from the 1000 Genomes Project, representing nine populations from Europe, Asia, Africa and the Americas. The dose response profile data from this study consists of the cytotoxic response to 179 environmental chemicals each assayed at 8 different concentrations, with 1.3 million SNPs used in the association analysis. Chemicals and concentrations used in this study are listed in Table S2. Cell lines were divided into screening batches equally distributed by population and sex in each batch to limit the effect of any systematic bias, such as that of population substructure, on the assay results. Each cell line was plated on one or two, 1,536-well plates with 2,000 cells/5µL/well. DMSO at 0.46% vol/vol was used as the negative control and tetra-octyl-ammonium bromide was used as the positive control. There were no significant differences in the control values between ethnic groups. The CellTiter-Glo Luminescent Cell Viability (Promega, Madison, WI, USA) assay was used to assess intracellular ATP concentration, a marker for viability/cytotoxicity, 40 h after treatment. Luminescent intensity was detected using a ViewLux plate reader (PerkinElmer). From the 1,086 cell lines, a subset of 884 “unrelated” individuals was chosen. Of the 884 individuals, genotyped SNPs were obtained from the Illumina HumanOmni2.5 platform for 761 individuals and from HapMap for the remaining 123 individuals. A set of 875 samples from the 1000 Genomes set were used as reference to impute 1.3 million SNPs for association analysis. Genotypic quality control on these SNPs resulted in 699,068 SNPs per chemical for association analysis.

The genomic/genotype data used for the chemical response is publicly available through the 1000 Genomes Project (http://www.internationalgenome.org). The genomic/genotype data used for the chemotherapy response can be found on dbGAP: Cholesterol and Pharmacogenetics (CAP) Study dbGaP Study Accession: phs000481.v2.p1.

**Figure 1:**
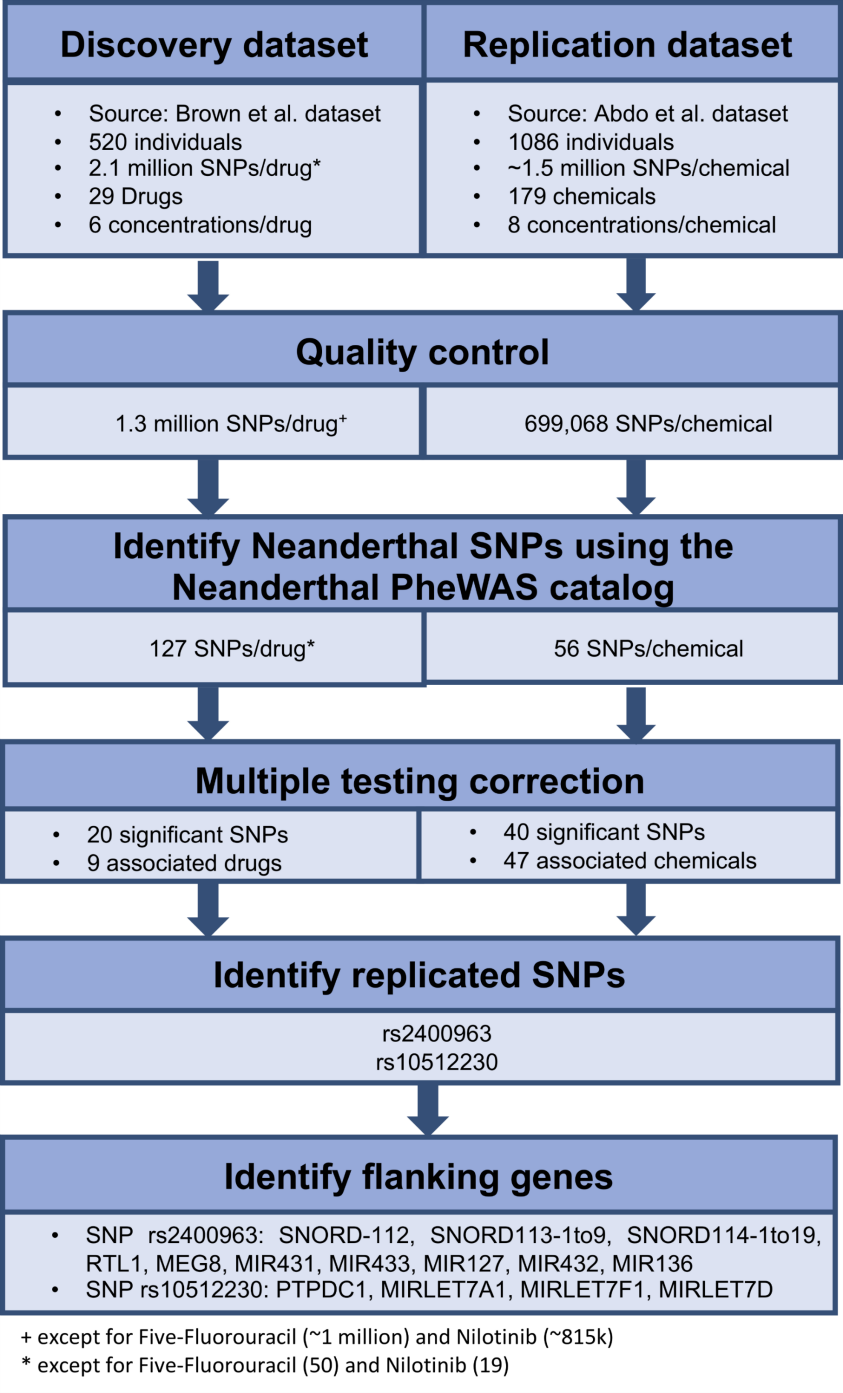
Workflow used to identify the effect of individual Neanderthal SNPs on dose response in the discovery and replication datasets.

We retrieved all the SNPs identified in each of the above genome-wide association mapping studies. We performed quality control on the data, which mainly involved removing rare variants, which was determined to be markers with <20 individuals for any genotype. We identified the Neanderthal-derived SNPs from the Neanderthal PheWAS catalog (Simonti et al., 2016) in each of the genome-wide data based on rs-ID. We then used these results as our initial data to identify the influence of individual Neanderthal-derived SNPs on dose response. A false discovery rate of *q* < 0.25, using the R function *p.adjust()* (package *stats v3.4.0*) (R Development Core Team, 2016), applied per drug or chemical was used to identify significant SNPs. For each of the significant Neanderthal SNPs that replicated in both datasets, we inspected a region of ±50 kilobases (kb) using LocusZoom (Pruim et al., 2010) (version 1.3, Human Genome assembly (build hg18) Lander et al., 2001) to locate flanking genes. The workflow for this analysis is depicted in Fig. 1.

**Figure 2:**
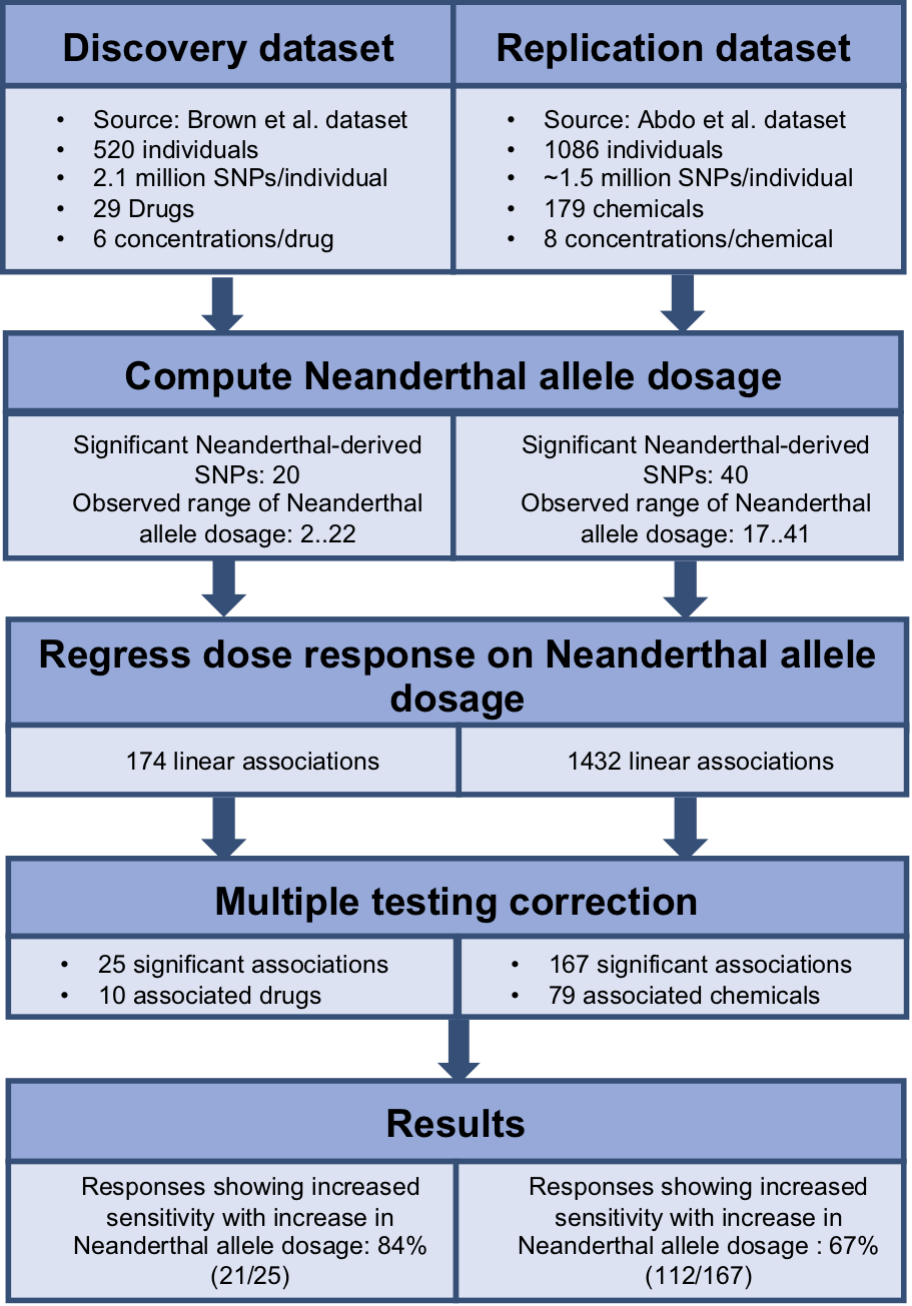
Workflow used to determine the cumulative effect of Neanderthal SNPs on dose response in the discovery and replication datasets.

To identify the cumulative effect of Neanderthal alleles on dose response, we performed burden tests. We used the dbSNP database (Sherry et al., 2001) to identify the Neanderthal allele for each of the significant Neanderthal-derived SNPs. We used PLINK v1.07 (Purcell; Purcell et al., 2007) to aggregate information across all significant Neanderthal-derived SNPs to compute a single Neanderthal allele dosage number representing the cumulative effect of these Neanderthal-derived SNPs in each individual, for each study. We then modeled each response, at each concentration, as a function of the Neanderthal allele dosage number using a linear regression approach (R function *lm()*, package *stats v3.4.0)* (R Development Core Team, 2016). Significant results were obtained after correcting for multiple testing, using the Benjamini–Hochberg method with a false discovery rate of *q* < 0.25, on a per drug or chemical basis (R function *p.adjust()*, package *stats v3.4.0*) (R Development Core Team, 2016). The workflow for this analysis is depicted in Fig. 2. We also performed burden testing using a univariate summary measure, the half maximal inhibitory concentration (EC_50_). We used linear regression to model EC_50_ as a function of the Neanderthal allele dosage number.

We attempted to perform phenome-wide association study (PheWAS)-based analyses to identify different phenotypes or phenotype groups associated with the Neanderthal-derived SNPs in our datasets as compared to the non-Neanderthal-derived SNPs. We isolated the phenotypes associated with the significant Neanderthal and non-Neanderthal SNPs in each of our datasets, using the PheWAS catalog (Denny et al., 2013). However, none or very few of the significant non-Neanderthal SNPs from the discovery and replication dataset were listed in the PheWAS catalog, thus preventing further analyses.

All data used in the study have been previously collected. All data are de-identified, and IRB exceptions have been filed.

## Results

### Effect of individual Neanderthal SNPs

Twenty Neanderthal-derived SNPs in the Brown et al. (2014) data and 40 Neanderthal-derived SNPs in the Abdo et al. (2015) data were deemed significant with a false discovery rate of *q* < 0.25 applied per drug or chemical. Two Neanderthal SNPs replicated in both data sets, rs2400963 and rs10512230. These significant replicated SNPs along with their flanking genes and the associated drugs or chemicals are listed in Table 1. The LocusZoom (Pruim et al., 2010) plots showing the regional genes for each SNP are shown in Figs. S1 and S2. All except the protein tyrosine phosphatase domain containing 1 (*PTPDC1*) gene are non-coding RNA genes. Several of the flanking genes have been previously shown to be associated with cancer. SNP rs2400963 is in the *SNORD113-3* gene. SNP rs2400963 and its flanking genes (*SNORD-112*, *SNORD113-1* to -*9*, *SNORD114-1* to -*19*, *RTL1*, *MEG8*, *MIR431* (*miR431*), *MIR433 (miR433)*, *MIR432 (miR432)*, *MIR136 (miR136)*) are located in the *DLK1-DIO3* locus at the *14q32* region. The *DLK1-DIO3* region contains several maternally and paternally expressed imprinted genes. This region hosts several miRNA clusters, many of which are differentially expressed in various cancers (Benetatos et al., 2013). For example, *MIR127* and other microRNA (miRNA) genes located at *14q32* region show overexpression in acute myeloid leukemia bearing a t(15;17) translocation (Valleron et al., 2012). Overexpression of the *DLK1-DIO3* miRNA cluster at *14q32.2* is associated with increased expression of hepatocellular carcinoma (HCC) stem cell markers and poor survival rate in HCC patients (Luk et al., 2011). A specific small nucleolar RNA (snoRNA) profile has been reported in acute promyelocytic leukemia (APL) with ectopic expression of *SNORD112*, *SNORD113* and *SNORD114* snoRNA clusters (Valleron et al., 2012). *SNORD113-1* has been shown to function as a tumor suppressor in HCC (Xu et al., 2014). SNP rs10512230 is an expression quantitative trait loci (eQTL) of the *RP11-307E17.8* gene (*LOC100132077*) while *RP11-307E17.8* is a non-coding RNA gene. Data in the Genotype-Tissue Expression (GTEX) Project (The GTEx Consortium, 2013) (accessed on 06/27/2017) shows that the Neanderthal allele at rs10512230 is significantly associated with increased expression of the *RP11-307E17.8* gene in transformed fibroblast cells and in the muscularis mucosae of the esophagus. SNP rs10512230 is flanked by the *PTPDC1*, *MIRLET7A1 (Let-7a-1)*, *MIRLET7F1 (Let-7f-1)* and *MIRLET7D* (*Let-7d*) genes. *PTPDC1* is a Protein Coding gene, in the p21-activated kinases (PAK) pathway. PAKs are involved in cell proliferation signaling, cell death resistance and metastasis, which are key hallmarks of cancer (Ye & Field, 2012). *MIRLET7A1*, *MIRLET7F1*, *MIRLET7D* are microRNA genes which are involved in cancer and DNA damage response pathways. Thus, as described above, various studies have reported links of the identified flanking genes in cancer pathways. Hence, we hypothesize that the Neanderthal-derived SNPs rs2400963 and rs10512230 may be associated with cytotoxic response.

**Table 1:** Individual Neanderthal SNPs with significant and replicating dose response associations along with their associated drug or chemical and flanking genes over a ±50 kb region. *SNORD*- small nucleolar RNA, C/D Box, *RTL1* retrotransposon Gag like 1, *MEG8*- maternally Expressed 8, *MIR* microRNA, *PTPDC1*- protein tyrosine phosphatase domain containing 1. Chromosomal position for SNPs was determined using the Human Genome assembly (build hg18). Multiple testing correction was done using the Benjamini-Hochberg method with a false discovery rate of *q* < 0.25, on a per drug or chemical basis.

**Chr:position**	**SNP**	**Drug/Chemical**	*p*-value	*q*-value	**Flanking genes**
14:100465459	rs2400963	Amiloride hydrochloride 2,3,4,5-Tetrachloronitrobenzene Nilotinib	3.97 × 10^−4^ 1.13 × 10^−3^ 1.04 × 10^−2^	2.22 × 10^−2^ 9.45 × 10^−2^ 1.98 × 10^−1^	*SNORD-112, SNORD113-1to9, SNORD114-1to19, RTL1, MEG8, MIR431, MIR433, MIR127, MIR432, MIR136*
9:95956051	rs10512230	Nitazoxanide Tetra-N-Octylammonium bromide 2-Biphenylamine Chlordane (technical grade) Cytarabine	2.58 × 10^−3^ 2.23 × 10^−3^ 8.07 × 10^−3^ 3.74 × 10^−3^ 1.21 × 10^−2^	1.45 × 10^−1^ 1.25 × 10^−1^ 2.40 × 10^−1^ 2.07 × 10^−1^ 2.21 × 10^−1^	*PTPDC1, MIRLET7A1, MIRLET7F1, MIRLET7D*

### Cumulative effect of Neanderthal alleles

We employed a linear regression model to analyze the cumulative effect of Neanderthal alleles on the dose–response phenotype, for each individual at each concentration for each drug or chemical. Each drug or chemical was considered as a separate test and a false discovery control was applied per drug or chemical. Results with false discovery rates of *q* < 0.25 were considered significant. Further, 25 Neanderthal allele dosage-cytotoxic response associations for 10 chemotherapy drugs from the Brown et al. (2014) data were declared significant. Also, 167 Neanderthal allele dosage-cytotoxic response associations for 79 environmental chemicals from the Abdo et al. (2015) data were declared significant.

**Figure 3:**
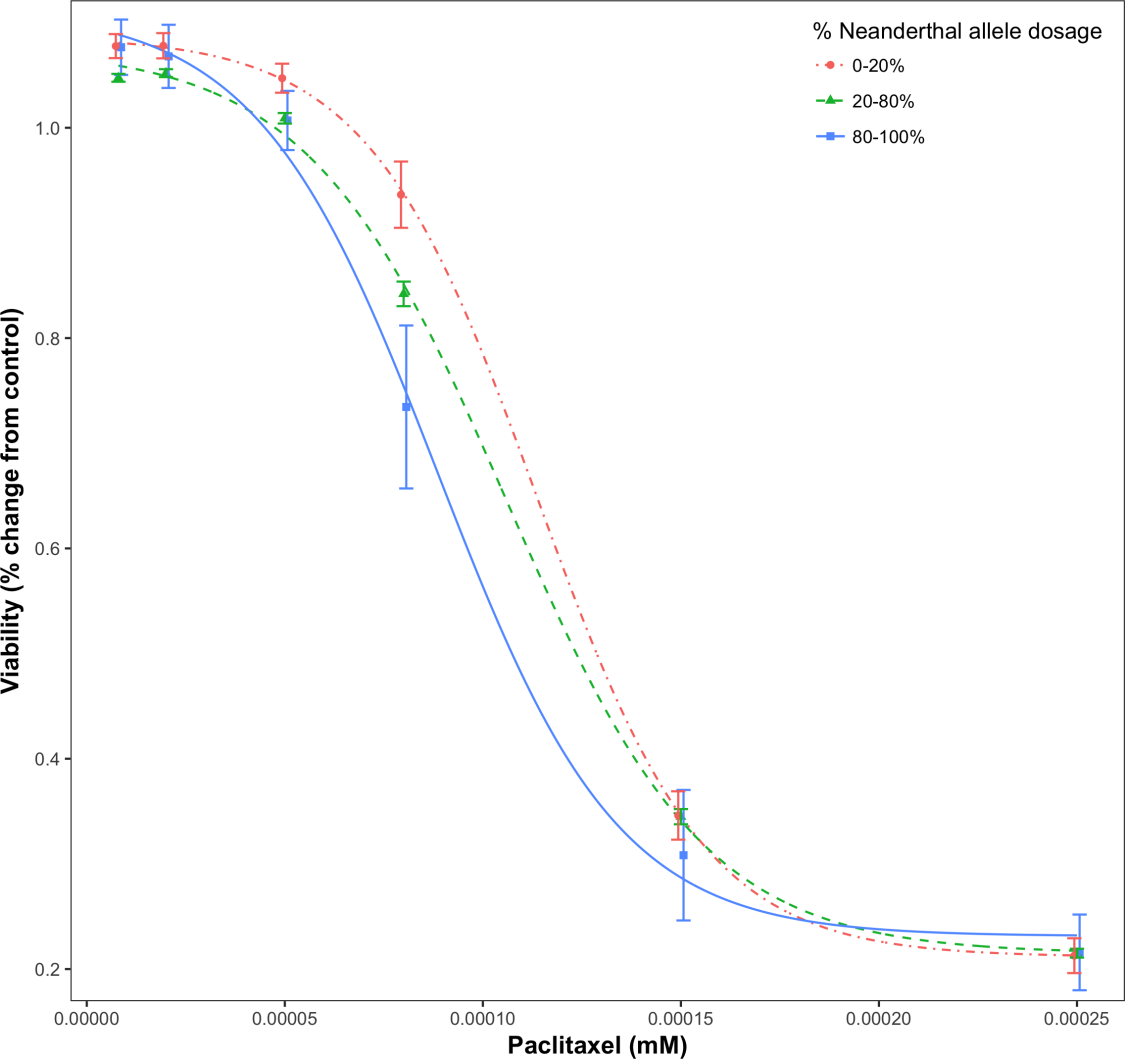
Dose-response curve for Paclitaxel, a chemotherapy drug. Dose-response is stratified by percent Neanderthal allele dosage as shown. Bars represent the standard error of the mean. Increase in Neanderthal allele dosage is accompanied by a decrease in cell viability at a fixed drug concentration.

For a given drug or chemical, at a fixed concentration, as the Neanderthal allele dosage increased, cell viability consistently decreased. This is shown in Fig. 3 for the chemotherapy drug Paclitaxel. In this test, 84% (21/25) of responses showed increased sensitivity to the chemotherapy drugs with increase in Neanderthal allele dosage. On the other hand, 67% (112/167) of responses showed increased susceptibility to environmental chemicals with increase in Neanderthal allele dosage. This is a remarkable trend observed in a large percentage of responses in data from both studies. Thus, we conclude that an increase in Neanderthal allele dosage leads to increased sensitivity to anti-cancer drugs and environmental chemicals.

We did not find consistent results in the linear regression model with EC_50_ as a function of the Neanderthal allele dosage number. This may be due to the fact that the univariate summary measure may not capture the true differences in the dose response profile if the differences are not entirely due to EC_50_. Univariate summary measures have been shown to be less robust and have less power as compared to the full dose–response profile (Brown et al., 2012b). Since the Abdo et al. (2015) data consists of cell lines from geographically and ancestrally diverse populations, we repeated the linear regression model for this data with the cell lines stratified by continental origin. Results from this stratified analysis showed the same trend as above for the cell lines from European and Asian populations but not for American and African populations. These results are as expected because the Eurasian genome contains the largest percentage of DNA inherited from Neanderthals (Sankararaman et al., 2012; Wall et al., 2013; Vernot & Akey, 2014). While the European population showed the trend of increased sensitivity with increase in Neanderthal allele dosage, we did observe fewer significant results in the European population than expected.

### Detailed results

In the main text we focus on the top results, but additional details of all the results can be found in the Supplemental Material. Table S3 includes details of all SNP-drug and SNP-chemical associations for the single SNP analysis, including the EC_50_ values for each of the genotypes. Table S4 expands on the hypothesis generation aspect of our analyses to provide additional annotation and references to support further investigation of putative associations.

## Discussion

Various studies have shown that archaic admixture has led to genetic variability that has played a role in human adaptation and continues to influence modern human traits. Denisovan introgression has been identified as the cause of an unusual haplotype in the *EPAS1* gene region leading to favorable altitude adaptation in Tibetans (Huerta-Sánchez et al., 2014). Neanderthal variants have been found to be associated with various modern human phenotypes, such as hypercoagulation, depression, actinic keratosis, urinary tract disorders, etc. (Simonti et al., 2016). Introgressed Neanderthal and Denisovan alleles were found to be associated with increased immune system response and increased susceptibility to allergic diseases (Dannemann, Andrés & Kelso, 2016).

However, to the best of our knowledge, the effect of introgressed Neanderthal alleles within the cell line model of drug response has not been studied. In this study, we demonstrate the influence of individual Neanderthal SNPs and cumulative Neanderthal allele dosage on cytotoxic response to 29 chemotherapy drugs and 179 environmental chemicals. Our results show another association between Neanderthal alleles and a clinically relevant modern human phenotype, cytotoxic response. Thus, our results provide additional insight into how Neanderthal introgression influences modern human traits. The cumulative effect of Neanderthal alleles on cytotoxic response is striking. Our results show that an increase in Neanderthal allele dosage is associated with an increased sensitivity to anti-cancer drugs and environmental chemicals. Of course, the response to anti-cancer agents and toxic chemicals is the measurable phenotype that associates with the residual Neanderthal variance in anatomically modern humans, but we hypothesize that Homo sapiens may have evolved resistance to a diverse array of phytochemicals due to a more heterogeneous diet relative to the predominantly meat-based diet of Neanderthals. Previous studies have shown the influence of race and ethnicity on the dose response phenotype (Shord et al., 2006; Sharma, Cogswell & Li, 2008; Fu et al., 2014; Abdo et al., 2015; Jack et al., 2015). In this study, we show a strong association between Neanderthal ancestry and the dose response phenotype. In a previous study consisting of patients with similar access to health care resources and treatment with adjuvant 5-fluorouracil for colon cancer, it was shown that individuals with Caucasian ancestry experienced higher treatment-related toxicity rates than individuals with African-American ancestry (McCollum et al., 2002). In another study from our group analyzing associations between race and variations in drug response to Paclitaxel in breast cancer patients (manuscript in preparation), we have found that at higher drug concentrations, cell lines from white individuals show a significant increase in sensitivity compared to cell lines from black individuals. This corroborates our results of increased sensitivity to cytotoxic chemicals with increased Neanderthal allele dosage, because the Caucasian genome contains a higher proportion of Neanderthal DNA than any other population, whereas the African genome contains very little to no Neanderthal DNA (Sankararaman et al., 2012; Wall et al., 2013; Vernot & Akey, 2014). Thus, the results from this study are consistent with the two studies described above, with all of them having the same direction of effect. Interestingly, the results of the Abdo et al. (2015) study showed that overall, Caucasian populations were less sensitive to environmental chemicals compared to the other global populations (the overall distribution of EC_10_ values for Caucasian populations is higher than for other populations). This reinforces our results in that while Caucasian populations have the overall highest Neanderthal allele dosage, our results are not confounded by the overall relative sensitivity of Caucasian populations.

While the cumulative effect of Neanderthal allele dosage is remarkable, there are some limitations that must be noted. In order to translate these results into a clinical setting, additional follow-up studies are needed, both related to understanding the biology of the result and to the translatability of the finding to patients. siRNA knock-down experiments, gene expression profiling assays and/or CRISPR/Cas9 gene editing techniques could be performed to validate the functional effects of Neanderthal alleles on cytotoxic response. The dose response profile data used in this study was assayed in LCLs, which have inherent limitations that must be addressed for the results of this study to be clinically relevant. Dose response is a complex trait and is influenced by various criteria such as absorption, distribution, metabolism, excretion and toxicity (ADMET). The LCL dose response assay is a measure of cellular toxicity which is only one of the many components of dose response. Thus, the LCL dose response does not completely represent an individual’s complex response to a chemical or drug. This limitation could be addressed by replicating this study in data from a clinical trial on cancer patients undergoing chemotherapy treatment to examine if Neanderthal alleles have an analogous effect on clinical outcome. In this study, we examined the influence of Neanderthal ancestry on the dose response phenotype; it would be interesting to inspect if Denisovan ancestry also has a similar influence on cytotoxic response.

## Conclusion

This study establishes that Neanderthal ancestry-informative markers (AIMs) do have an influence on cytotoxic response, which is a clinically important trait. We have identified Neanderthal-derived SNPs that could be associated with cytotoxic response and showed that an increase in Neanderthal allele dosage leads to increased sensitivity to anti-cancer drugs and environmental chemicals. The results from this study shed light on another phenotype influenced by Neanderthal DNA. The findings in this study provide another dimension of the genetic factors that influence how an individual or a population will respond to a drug or a chemical, which is a key goal of pharmacogenomics and toxicogenomics.

##  Supplemental Information

10.7717/peerj.5691/supp-1 Supplemental Information 1 Supplemental Information **Figure S1.** LocusZoom plot showing the regional genes for SNP rs2400963 over a region of 50 kilobase pairs.
**Figure S2.** LocusZoom plot showing the regional genes for SNP rs10512230 over a region of 50 kilobase pairs.
**Table S1.** List of anti-cancer drugs and their concentrations used in the genome-wide association mapping of cytotoxic response in LCLs derived from unrelated Caucasian individuals from the Children’s Hospital of Oakland Research Institute (CHORI).
**Table S2.** List of environmental chemicals and their concentrations used in the genome-wide association mapping of cytotoxic response in LCLs from the 1000 Genomes (1000G) Project.
**Table S3.**List of all significant SNP-drug or SNP-chemical associations and their median EC_50_ value for each genotype.

10.7717/peerj.5691/supp-2 Supplemental Information 2 Table S4. Annotated information of putative SNP-drug and SNP-chemical associations

## Additional Information and Declarations

### Data Availability

The following information was supplied regarding data availability: The research in this article did not generate any data or code. All data used in the study had been previously collected. All data are de-identified, and IRB exceptions have been filed. The genomic/genotype data used for the chemical response is publicly available through the 1000 Genomes Project, and can be found here: http://www.internationalgenome.org. The genomic/genotype data used for the chemotherapy response can be found on dbGAP: Cholesterol and Pharmacogenetics (CAP) Study. dbGaP Study Accession: phs000481.v2.p1.
