# Supplementary material for: The influence of Neanderthal alleles on cytotoxic response
Source: PeerJ. 2018 Oct 23;6:e5691. doi: 10.7717/peerj.5691 (PMC6202974; doi:10.7717/peerj.5691)
Supplement: Supplemental Information 1 — Figure S1. LocusZoom plot showing the regional genes for SNP rs2400963 over a region of 50 kilobase pairs. Figure S2. LocusZoom plot showing the regional genes for SNP rs10512230 over a region of 50 kilobase pairs. Table S1. List of anti-cancer drugs and their concentrations used in the genome-wide association mapping of cytotoxic response in LCLs derived from unrelated Caucasian individuals from the Children’s Hospital of Oakland Research Institute (CHORI). Table S2. List of environmental chemicals and their concentrations used in the genome-wide association mapping of cytotoxic response in LCLs from the 1000 Genomes (1000G) Project. Table S3.List of all significant SNP-drug or SNP-chemical associations and their median EC50 value for each genotype. [file peerj-06-5691-s001.docx]

**Supplemental Material**

**Figure S1.** LocusZoom plot showing the regional genes for SNP *rs2400963* over a region of 50 kilobase pairs.

**Figure S2.** LocusZoom plot showing the regional genes for SNP *rs10512230* over a region of 50 kilobase pairs.

**Table S1.** List of anti-cancer drugs and their concentrations used in the genome-wide association mapping of cytotoxic response in LCLs derived from unrelated Caucasian individuals from the Children’s Hospital of Oakland Research Institute (CHORI). Conc = concentrations in mM.

| **Drug** | **Conc1** | **Conc2** | **Conc3** | **Conc4** | **Conc5** | **Conc6** |
| --- | --- | --- | --- | --- | --- | --- |
| ArsenicTrioxide | 0.02 | 0.01 | 0.008 | 0.004 | 0.002 | 0.001 |
| Azacitidine | 0.12 | 0.08 | 0.06 | 0.04 | 0.01 | 0.005 |
| Carboplatin | 0.06 | 0.04 | 0.02 | 0.01 | 0.008 | 0.004 |
| Cladribine | 0.4 | 0.12 | 0.04 | 0.02 | 0.008 | 0.004 |
| Cytarabine | 0.01 | 0.005 | 0.0025 | 0.0014 | 8.00E-04 | 4.00E-04 |
| Dasatinib | 0.09 | 0.05 | 0.01 | 0.005 | 1.00E-04 | 1.00E-05 |
| Daunorubicin | 1.00E-04 | 8.00E-05 | 5.00E-05 | 2.50E-05 | 1.00E-05 | 5.00E-06 |
| Docetaxel1 | 2.00E-04 | 0.00015 | 1.00E-04 | 8.00E-05 | 6.00E-05 | 2.00E-05 |
| Docetaxel2 | 6.80E-05 | 5.70E-05 | 4.80E-05 | 4.00E-05 | 3.30E-05 | 2.00E-05 |
| Doxorubicin | 0.00025 | 1.00E-04 | 7.50E-05 | 5.00E-05 | 1.30E-05 | 6.00E-06 |
| Epirubicin | 6.00E-04 | 4.00E-04 | 2.00E-04 | 8.00E-05 | 4.00E-05 | 2.00E-05 |
| Etoposide | 0.0075 | 0.0025 | 0.0015 | 0.00075 | 5.00E-04 | 0.00025 |
| 5-Fluorouracil | 0.25 | 0.1 | 0.05 | 0.02 | 0.005 | 0.001 |
| Floxuridine | 0.8 | 0.2 | 0.1 | 0.002 | 2.00E-04 | 2.00E-05 |
| Fludarabine | 0.25 | 0.15 | 0.12 | 0.067 | 0.05 | 0.025 |
| Gemcitabine | 1.00E-04 | 4.00E-05 | 2.00E-05 | 8.00E-06 | 5.00E-06 | 2.50E-06 |
| Hydroxyurea | 0.4 | 0.3 | 0.2 | 0.15 | 0.075 | 0.025 |
| Idarubicin | 0.00012 | 8.00E-05 | 4.00E-05 | 3.00E-05 | 2.00E-05 | 1.00E-05 |
| Mitomycin | 0.001 | 5.00E-04 | 3.00E-04 | 2.00E-04 | 8.00E-05 | 2.50E-05 |
| Mitoxantrone | 5.00E-04 | 2.00E-04 | 1.00E-04 | 4.00E-05 | 2.00E-05 | 8.00E-06 |
| Nilotinib | 0.0625 | 0.05 | 0.0375 | 0.02917 | 0.01 | 0.005 |
| Oxaliplatin | 0.02 | 0.01 | 0.005 | 0.003 | 0.0015 | 5.00E-04 |
| Paclitaxel | 0.00025 | 0.00015 | 8.00E-05 | 5.00E-05 | 2.00E-05 | 8.00E-06 |
| Sunitinib | 0.01 | 0.008 | 0.00625 | 0.0042 | 0.0021 | 4.00E-04 |
| Temozolomide | 2.5 | 2 | 1 | 0.5 | 0.25 | 0.1 |
| Teniposide | 0.002 | 0.00025 | 1.00E-04 | 5.00E-05 | 1.00E-05 | 5.00E-06 |
| Topotecan | 5.00E-05 | 4.00E-05 | 3.00E-05 | 2.00E-05 | 1.00E-05 | 8.00E-06 |
| Vinblastine | 6.00E-05 | 2.50E-05 | 2.00E-05 | 4.00E-06 | 1.00E-06 | 6.00E-07 |
| Vincristine | 4.00E-05 | 1.00E-05 | 8.00E-06 | 6.00E-06 | 4.00E-06 | 2.00E-06 |
| Vinorelbine | 7.50E-05 | 5.50E-05 | 4.50E-05 | 3.50E-05 | 2.50E-05 | 1.90E-05 |

**Table S2.** List of environmental chemicals and their concentrations used in the genome-wide association mapping of cytotoxic response in LCLs from the 1000 Genomes (1000G) Project. The eight concentrations for each drug ranged from 0.33 nM to 92 μM.

| o-Phenanthroline | 2,4-Hexadienal | Diethylene glycol diacrylate | m-Nitrobenzyl chloride |
| --- | --- | --- | --- |
| t-Butylhydroquinone | Aldicarb | 1,8-Dihydroxy-4,5-dinitroanthraquinone | o-Nitrobenzyl chloride |
| t-Butylhydroquinone | p-Aminophenol | 3,4-Dinitrotoluene | Bis(cyclopentadienyl)vanadium chloride |
| Azathioprine | 13-cis-Retinal | Fumaronitrile | 2,3,5-Trichlorophenol |
| Amiloride hydrochloride | Retinal | HC blue 2 | 1,2-Epoxy-3-chloropropane (Epichlorohydrin) |
| Amitriptyline HCl | 2,3,4,5-Tetrachloronitrobenzene | Chloranil | Diglycidyl resorcinol ether (DGRE) |
| p-Quinone | Alizarin Yellow R, free acid | 1,6-Hexamethylene diacrylate | N,N'-Di-sec-butyl-p-phenyldiamine |
| Dexamethazone | Tetrachlorvinphos | 2,2',4'-Trichloroacetophenone | 4-Amino-4'-hydroxy-3-methyl-diphenylamine |
| Chlorambucil | Methyl mercuric (II) chloride | 3,4-Diaminotoluene | N-Isopropyl-N'-phenyl-p-phenylenediamine |
| Catechol | 2-Pivalyl-1,3-indandione | Hexachloro-1,3-butadiene | 2,3,4,5-Tetrachlorophenol |
| 17beta-Estradiol | N,N'-Diphenyl-p-phenylenediamine | 4-Chloro-3,5-dinitro-a,a,a-trifluorotoluene | Nitrogen mustard hydrochloride |
| 5-Fluorouracil | 6-Thioguanine (6-TG) | p-Nitrosodiphenylamine | 4-Methoxy-3-nitro-N-phenylbenzamide |
| Flutamide (pubertal study) | o-Phenylenediamine | N,N-Diethyl-p-phenylenediamine | 2',4',5'-Trihydroxybutyrophenone |
| Hydroquinone | p-n-Nonylphenol | Dieldrin | 1-(2,6,6-Trimethyl-2-cyclohexene-1-yl)-1-penten-3-one |
| Melatonin | Cadmium chloride | Diisobutyl phthalate | Azobenzene |
| Progesterone | 1,3-Diiminobenz (f)-isoindoline | Danthron | 2-Octyl-3-isothiazolone |
| Progesterone | 3,4-Dichlorophenyl isocyanate | N-Methyl-p-aminophenol sulfate | Toxaphene |
| Reserpine | dimethyldipropylene-triamine | 4-(Chloroacetyl)acetanilide | Aldrin |
| Triamterene | 2,4-Difluoronitrobenzene | Ethidium bromide | Sodium dichromate dihydrate (VI) |
| Verapamil HCl | Dichlorvos (Vapona) | Dibromoacetonitrile | Sodium dichromate dihydrate (VI) |
| Chlorhexidine | Chlordecone (kepone) | Pentaerythritol triacrylate | N-(1-Naphthyl) ethylenediamine dihydrochloride |
| Pyrimethamine | Chlordecone (kepone) | Chlordane (technical grade) | Chlorpheniramine maleate |
| Ethacrynic acid | Domiphen bromide | 1-Methyl-3-nitro-1-nitroso-guanidine | Malachite green oxalate |
| Sulfathiazole | tetra-N-Octylammonium bromide | 1,1,1,2-Tetrabromoethane | Cadmium acetatedihydrate |
| Ethoxyquin | Cetylpyridinium bromide | p-tert-Butylcatechol | 9-Aminoacridine, monohydrochloride, monohydrate |
| Benzethonium chloride | Tetraethylene glycol diacrylate | Dibromonitromethane (water disinfection byproducts) | Colchicine |
| Iodochlorohydroxyquinoline | Tetraethylene glycol diacrylate | 1,3-Dicyclohexylcarbodiimide | Colchicine |
| Phenformin hydrochloride | Captan | Endosulfan | Hematoxylin |
| Turmeric (>98% curcurmin) | Captan | Di(2-ethylhexyl) phthalate | Propiconazole |
| 2,2'-Thiobis(4,6-dichlorophenol) | Phenylmercuric acetate | 4,4-Thiobis(6-tert-butyl-m-cresol) | Systhane |
| Rhein (1,8-dihydroxy-3-carboxyl anthraquinone) | Styrene | 7,12-Dimethylbenzanthracene | Ziram |
| Tamoxifen citrate | trans-1,4-dichloro-2-butene | Methylene bis(thiocyanate) | Cycloheximide |
| Bisphenol A diglycidyl ether | Ethyl linolenate | 5-(Hydroxymethyl)-2-furoic acid | Cycloheximide |
| Dazomet | 1-Naphthylamine | N-(1,3-Dimethylbutyl)-N'-phenyl-p-phenylenediamine | Aflatoxin B1 from Aspergillus flavus |
| 2,4-Decadienal | beta-Nitrostyrene | Tetramethylthiouram disulfide | permethrin |
| 8-Hydroxyquinoline | Glutaraldehyde | 2-Amino-4-chlorophenol | Guggulsterones E |
| 8-Hydroxyquinoline | Hexamethyl-p-rosaniline chloride | Mercuric chloride | Daunomycin HCL |
| 3-Chloro-4-(dichloromethyl)-5-hydroxy-2(5H)-furanone(MX) | Ninhydrin | 2-Amino-4-methylphenol | Saquinavir mesylate (AIDS Initiative) |
| Methacrylonitrile | dichloroacetonitrile | Titanocene dichloride | Oxymetholone |
| Acetochlor | 2-Biphenylamine | 6-Mercaptopurine monohydrate | Digoxin |
| Potassium dichromate | N,N,N',N'-Tetramethyl-p-phenylenediamine | p-Nitrophenethyl alcohol | p-Benzoquinone dioxime |
| Retinol acetate | 1,3,5-Triglycidyl isocyanurate | 4-Chloro-o-phenylenediamine | Vitamin D3 |
| cis-Dichlorodiamine platinum | o-Aminophenol | t-Butyl formate | Zinc pyrithione |
| Mono(2-ethylhexyl)phthalate | Hexachlorophene | N,N-Dimethyl-p-nitrosoaniline | Ergotamine tartrate |
| Nitazoxanide | 2-Chloroacetophenone (CN) | Nifedipine |  |

**Table S3.** List of all significant SNP-drug or SNP-chemical associations and their median EC_50_ value for each genotype. The EC_50_ values for the chemotherapy response from the Brown et al. dataset, were computed from curve fitting the dose response data using the Evolutionary Algorithm Dose Response Modeling (EADRM) software (Beam AL, Motsinger-Reif AA., 2011, *Dose-Response*). The EC_50_ values reported here are raw values obtained from curve fitting and have not been corrected or characterized for variability or issues of extrapolation. The EC_50_ values for the chemical response from the Abdo et al. dataset, were computed as described in Abdo et al., 2015, *Environmental Health Perspectives*. Hu/Hu – Human/Human, Hu/Nt – Human/Neanderthal, Nt/Nt – Neanderthal/Neanderthal.

|  |  |  | **Median EC_50_ value for each genotype** | | |
| --- | --- | --- | --- | --- | --- |
| **Chr** | **SNP** | **Drug/Chemical name** | **Hu/Hu** | **Hu/Nt** | **Nt/Nt** |
| 1 | rs10798918 | Methacrylonitrile | 1.48E-01 | 2.63E-01 | 4.18E-01 |
| 1 | rs10798918 | N-(1,3-Dimethylbutyl)-N'-phenyl-p-phenylenediamine | 4.23E+01 | 4.43E+01 | 4.19E+01 |
| 1 | rs10798918 | Toxaphene | 4.43E+01 | 4.43E+01 | 4.18E+01 |
| 1 | rs10915535 | p-tert-Butylcatechol | 3.57E+01 | 3.73E+01 | 3.72E+01 |
| 1 | rs10915535 | tetra-N-Octylammonium bromide | 1.98E+00 | 2.10E+00 | 2.31E+00 |
| 1 | rs10915535 | Ziram | 2.09E-01 | 2.63E-01 | 2.52E-01 |
| 1 | rs12028524 | 2-Octyl-3-isothiazolone | 4.68E+00 | 4.97E+00 | 4.68E+00 |
| 1 | rs12028524 | 5-Fluorouracil | 4.48E+00 | 3.70E+00 | 5.65E+00 |
| 1 | rs12028524 | tetra-N-Octylammonium bromide | 2.35E+00 | 2.09E+00 | 2.09E+00 |
| 1 | rs1361730 | 9-Aminoacridine, monohydrochloride, monohydrate | 4.68E+00 | 4.68E+00 | 4.97E+00 |
| 1 | rs1361730 | Di(2-ethylhexyl) phthalate | 2.35E+00 | 5.26E+00 | 5.91E+00 |
| 1 | rs16835617 | Hydroquinone | 3.32E+01 | 3.32E+01 | 2.91E+01 |
| 1 | rs2296172 | 4-(Chloroacetyl)acetanilide | 4.43E+01 | 4.29E+01 | 4.18E+01 |
| 1 | rs2296172 | Malachite green oxalate | 7.02E-02 | 7.42E-02 | 9.35E-02 |
| 1 | rs2296172 | Nifedipine | 3.72E+01 | 3.75E+01 | 3.72E+01 |
| 1 | rs2296173 | 4-(Chloroacetyl)acetanilide | 4.43E+01 | 4.24E+01 | 4.18E+01 |
| 1 | rs2296173 | Malachite green oxalate | 7.02E-02 | 7.42E-02 | 9.35E-02 |
| 1 | rs2296173 | Nifedipine | 3.72E+01 | 3.79E+01 | 3.72E+01 |
| 1 | rs3765865 | 2,3,5-Trichlorophenol | 4.18E+01 | 4.08E+01 | 3.82E+01 |
| 1 | rs3765865 | Oxymetholone | 4.68E+01 | 4.68E+01 | 4.97E+01 |
| 1 | rs4844488 | 2,3,4,5-Tetrachloronitrobenzene | 3.72E+01 | 3.72E+01 | 3.84E+01 |
| 1 | rs4844488 | Chlorpheniramine maleate | 3.00E+01 | 3.13E+01 | 3.63E+01 |
| 2 | rs3771635 | 2-Amino-4-chlorophenol | 2.35E+01 | 2.22E+01 | 2.37E+01 |
| 2 | rs3771635 | 2,4-Decadienal | 3.72E+01 | 3.72E+01 | 4.16E+01 |
| 2 | rs3771635 | Diisobutyl phthalate | 4.73E-02 | 2.11E-01 | 1.45E-01 |
| 2 | rs3771635 | Malachite green oxalate | 7.42E-02 | 6.26E-02 | 7.05E-02 |
| 2 | rs3771635 | Methacrylonitrile | 1.32E-01 | 4.37E-01 | 4.18E-01 |
| 3 | rs12638360 | 5-Fluorouracil | 4.68E+00 | 5.29E+00 | 1.63E+00 |
| 3 | rs12638360 | t-Butyl formate | 3.32E+00 | 3.32E+00 | 3.61E+00 |
| 3 | rs13097326 | 7,12-Dimethylbenzanthracene | 3.80E+01 | 3.75E+01 | 3.72E+01 |
| 3 | rs1566479 | Titanocene dichloride | 4.18E+01 | 4.00E+01 | 3.51E+01 |
| 3 | rs5186 | 7,12-Dimethylbenzanthracene | 3.75E+01 | 3.84E+01 | 3.72E+01 |
| 3 | rs5186 | Nifedipine | 3.72E+01 | 3.72E+01 | 4.18E+01 |
| 3 | rs9855367 | Doxorubicin | 3.87E-05 | 4.00E-05 | 3.71E-05 |
| 4 | rs13130029 | Cytarabine | 4.30E-03 | 3.51E-03 | 1.58E-03 |
| 4 | rs13130029 | Sunitinib | 3.08E-03 | 3.03E-03 | 2.79E-03 |
| 4 | rs7687260 | Mitoxantrone | 3.08E-05 | 2.98E-05 | 2.65E-05 |
| 5 | rs17428724 | Cladribine | 1.04E-02 | 1.44E-02 | 1.40E-02 |
| 5 | rs17428724 | Cytarabine | 3.96E-03 | 3.99E-03 | 3.97E-03 |
| 5 | rs17618372 | cis-Dichlorodiamine platinum | 3.32E+01 | 3.08E+01 | 2.63E+01 |
| 5 | rs2304033 | Cladribine | 1.04E-02 | 1.47E-02 | 1.44E-02 |
| 5 | rs2304033 | Cytarabine | 3.92E-03 | 4.47E-03 | 3.61E-03 |
| 5 | rs3749994 | Cladribine | 1.04E-02 | 1.43E-02 | 1.44E-02 |
| 5 | rs3749994 | Cytarabine | 3.96E-03 | 3.98E-03 | 4.25E-03 |
| 5 | rs7443214 | Cladribine | 1.06E-02 | 1.46E-02 | 1.40E-02 |
| 6 | rs10455593 | Sunitinib | 3.06E-03 | 3.09E-03 | 2.87E-03 |
| 6 | rs10457421 | t-Butyl formate | 3.72E+00 | 3.13E+00 | 3.18E+00 |
| 6 | rs12201990 | Ninhydrin | 4.49E+01 | 4.82E+01 | 5.04E+01 |
| 6 | rs12201990 | p-tert-Butylcatechol | 3.72E+01 | 3.54E+01 | 3.75E+01 |
| 6 | rs12201990 | t-Butyl formate | 2.39E+00 | 2.89E+00 | 3.72E+00 |
| 6 | rs12209650 | Sunitinib | 3.04E-03 | 3.03E-03 | 3.37E-03 |
| 6 | rs1563929 | Sunitinib | 3.03E-03 | 3.16E-03 | 2.63E-03 |
| 6 | rs4714738 | N,N'-Di-sec-butyl-p-phenyldiamine | 6.51E+00 | 5.26E+00 | 4.97E+00 |
| 8 | rs17079571 | Diglycidyl resorcinol ether (DGRE) | 1.40E+01 | 1.32E+01 | 1.32E+01 |
| 9 | rs10512230 | 2-Biphenylamine | 8.33E+00 | 1.18E+01 | 9.38E+00 |
| 9 | rs10512230 | Chlordane (technical grade) | 4.68E+01 | 4.68E+01 | 4.56E+01 |
| 9 | rs10512230 | Cytarabine | 4.25E-03 | 3.99E-03 | 2.95E-03 |
| 9 | rs10512230 | Nitazoxanide | 3.32E+01 | 2.54E+01 | 2.91E+01 |
| 9 | rs10512230 | tetra-N-Octylammonium bromide | 2.30E+00 | 2.22E+00 | 2.30E+00 |
| 9 | rs10978949 | Sunitinib | 3.09E-03 | 3.03E-03 | 2.47E-03 |
| 9 | rs10981252 | 2-Amino-4-chlorophenol | 2.35E+01 | 2.22E+01 | 2.49E+01 |
| 9 | rs17355458 | Cytarabine | 3.93E-03 | 4.06E-03 | 4.05E-03 |
| 9 | rs7039165 | Oxymetholone | 4.68E+01 | 4.68E+01 | 4.68E+01 |
| 9 | rs7850580 | Cytarabine | 3.96E-03 | 4.04E-03 | 4.05E-03 |
| 10 | rs11248442 | o-Phenylenediamine | 4.43E+01 | 3.06E+01 | 3.03E+01 |
| 10 | rs2453 | 2-Amino-4-chlorophenol | 2.09E+01 | 2.56E+01 | 2.23E+01 |
| 10 | rs2453 | Methacrylonitrile | 1.86E+00 | 1.05E-01 | 2.09E-01 |
| 10 | rs2453 | tetra-N-Octylammonium bromide | 2.16E+00 | 2.36E+00 | 2.22E+00 |
| 10 | rs2453 | Ziram | 2.10E-01 | 2.63E-01 | 2.51E-01 |
| 11 | rs10892201 | Hydroquinone | 3.11E+01 | 3.35E+01 | 3.32E+01 |
| 11 | rs12790857 | Temozolomide | 4.48E-01 | 4.45E-01 | 4.56E-01 |
| 11 | rs2156528 | 4,4-Thiobis(6-tert-butyl-m-cresol) | 2.96E+01 | 2.96E+01 | 3.14E+01 |
| 11 | rs2156528 | trans-1,4-dichloro-2-butene | 4.16E+00 | 3.31E+00 | 2.63E+00 |
| 11 | rs2307073 | Hematoxylin | 3.95E+01 | 4.18E+01 | 4.56E+01 |
| 11 | rs2307073 | Iodochlorohydroxyquinoline | 3.09E+01 | 3.28E+01 | 3.32E+01 |
| 11 | rs2307073 | t-Butyl formate | 3.01E+00 | 4.75E+00 | 1.36E+01 |
| 11 | rs7110434 | Temozolomide | 4.51E-01 | 4.45E-01 | 4.15E-01 |
| 12 | rs11049548 | 5-Fluorouracil | 4.55E+00 | 5.14E+00 | 9.32E+00 |
| 12 | rs12423929 | Pyrithione zirconium | 1.35E+00 | 1.44E+00 | 9.92E-01 |
| 12 | rs12427160 | Chlordane (technical grade) | 4.68E+01 | 4.68E+01 | 4.30E+01 |
| 12 | rs12427160 | Ninhydrin | 5.04E+01 | 5.04E+01 | 4.30E+01 |
| 12 | rs12811504 | 2-Amino-4-chlorophenol | 2.35E+01 | 2.33E+01 | 2.22E+01 |
| 12 | rs12811504 | 3,4-Dinitrotoluene | 3.32E+01 | 3.32E+01 | 3.31E+01 |
| 12 | rs12811504 | 6-Thioguanine (6-TG) | 4.97E+00 | 5.26E+00 | 3.50E+00 |
| 12 | rs12811504 | Diisobutyl phthalate | 5.90E-02 | 1.58E-01 | 1.57E-02 |
| 12 | rs12811504 | Hematoxylin | 3.95E+01 | 3.95E+01 | 4.18E+01 |
| 12 | rs12811504 | Oxymetholone | 4.68E+01 | 4.68E+01 | 4.68E+01 |
| 12 | rs12811504 | t-Butyl formate | 3.32E+00 | 3.73E+00 | 1.48E+00 |
| 12 | rs12811504 | Ziram | 2.56E-01 | 2.35E-01 | 2.63E-01 |
| 12 | rs2200275 | 2-Biphenylamine | 9.55E+00 | 1.12E+01 | 1.11E+01 |
| 12 | rs2200275 | Chlordecone (kepone) | 4.00E+01 | 3.95E+01 | 4.18E+01 |
| 12 | rs2200275 | p-tert-Butylcatechol | 3.72E+01 | 3.75E+01 | 3.92E+01 |
| 12 | rs4761177 | Mitoxantrone | 2.94E-05 | 3.07E-05 | 3.34E-05 |
| 12 | rs7979823 | Mitoxantrone | 2.94E-05 | 3.07E-05 | 3.34E-05 |
| 13 | rs16971270 | Chlordecone (kepone) | 4.00E+01 | 4.00E+01 | 4.18E+01 |
| 13 | rs16971270 | p-Benzoquinone dioxime | 4.18E+01 | 4.18E+01 | 4.13E+01 |
| 13 | rs9316483 | 1,8-Dihydroxy-4,5-dinitroanthraquinone | 2.83E+01 | 2.80E+01 | 3.32E+01 |
| 14 | rs2400963 | 2,3,4,5-Tetrachloronitrobenzene | 3.72E+01 | 3.74E+01 | 3.72E+01 |
| 14 | rs2400963 | Amiloride hydrochloride | 3.40E+01 | 3.42E+01 | 3.52E+01 |
| 14 | rs2400963 | Nilotinib | 1.28E-02 | 7.40E-03 | 8.11E-03 |
| 14 | rs2886122 | 5-Fluorouracil | 3.17E+00 | 7.47E+00 | 4.18E+00 |
| 14 | rs2886122 | Methacrylonitrile | 2.11E-01 | 1.66E-01 | 1.68E-01 |
| 14 | rs2886122 | Methylene bis(thiocyanate) | 8.77E+00 | 7.42E+00 | 7.88E+00 |
| 14 | rs2886122 | Tetraethylene glycol diacrylate | 2.09E+01 | 2.87E+01 | 2.63E+01 |
| 14 | rs2886122 | Ziram | 2.35E-01 | 2.13E-01 | 2.59E-01 |
| 16 | rs10514412 | t-Butyl formate | 3.24E+00 | 4.57E+00 | 6.26E+00 |
| 16 | rs4784260 | ArsenicTrioxide | 1.36E-02 | 1.39E-02 | 1.77E-02 |
| 16 | rs4784260 | Azacitidine | 2.71E-02 | 2.87E-02 | 3.26E-02 |
| 16 | rs4784260 | Sunitinib | 2.94E-03 | 3.16E-03 | 3.96E-03 |
| 16 | rs4887941 | Dichlorvos (Vapona) | 3.52E+01 | 3.39E+01 | 2.63E+01 |
| 17 | rs4968063 | N-(1-Naphthyl)ethylenediamine dihydrochloride | 4.43E+01 | 4.43E+01 | 4.43E+01 |
| 17 | rs4968063 | tetra-N-Octylammonium bromide | 2.35E+00 | 2.09E+00 | 1.98E+00 |
