# Supplementary material for: The influence of Neanderthal alleles on cytotoxic response
Source: PeerJ. 2018 Oct 23;6:e5691. doi: 10.7717/peerj.5691 (PMC6202974; doi:10.7717/peerj.5691)
Supplement: Supplemental Information 2 [file peerj-06-5691-s002.docx]

**Table S4**. List of all significant SNP-drug and SNP-chemical associations from the individual SNP analysis along with their flanking genes over a +/-50kb region, target eQTL genes and additional annotation and references to support further investigation of putative associations. The eQTL genes were retrieved from the Genotype-Tissue Expression (GTEX) Project (GTExC, 2013) (accessed on 4/19/2018). Multiple testing correction was performed to obtain q-values using the Benjamini-Hochberg method with a false discovery rate of q <0.25, on a per drug or chemical basis.

| **Chr** | **SNP** | **Drug/Chemical** | **p-value** | **q-value** | **Flanking genes** | **eQTL genes** | **Annotations for putative Neanderthal allele -cytotoxicity associations** | **References** |
| --- | --- | --- | --- | --- | --- | --- | --- | --- |
| 1 | rs10798918 | Methacrylonitrile | 1.68E-03 | 9.39E-02 | NA | FNDC5, TMEM54, KIAA1522, S100PBP, FKSG48, YARS | The Neanderthal allele for rs10798918 decreases S100PBP expression in lung tissue and transformed fibroblasts. S100PBP has been shown to be up-regulated in pancreatic ductal adenocarcinoma and may play an important role in the adhesive, migratory and invasive capabilities of pancreatic cancer cells. | Greenberg et al., 2007; Zeng et al., 2010; Lines et al., 2012; Dowen et al., 2005; |
|  |  | N-(1,3-Dimethylbutyl)-N'-phenyl-p-phenylenediamine | 2.55E-03 | 1.43E-01 |  |  |  |  |
|  |  | Toxaphene | 4.56E-04 | 2.55E-02 |  |  |  |  |
| 1 | rs10915535 | p-tert-Butylcatechol | 1.02E-02 | 2.43E-01 | NA | NA | NA |  |
|  |  | tetra-N-Octylammonium bromide | 1.45E-02 | 1.63E-01 |  |  |  |  |
|  |  | Ziram | 9.72E-03 | 1.81E-01 |  |  |  |  |
| 1 | rs12028524 | 2-Octyl-3-isothiazolone | 3.28E-03 | 1.84E-01 | NA | NA | NA |  |
|  |  | 5-Fluorouracil | 6.92E-03 | 1.82E-01 |  |  |  |  |
|  |  | tetra-N-Octylammonium bromide | 1.02E-02 | 1.63E-01 |  |  |  |  |
| 1 | rs1361730 | 9-Aminoacridine, monohydrochloride, monohydrate | 2.40E-03 | 1.34E-01 | NA | NA | NA |  |
|  |  | Di(2-ethylhexyl) phthalate | 1.50E-03 | 8.41E-02 |  |  |  |  |
| 1 | rs16835617 | Hydroquinone | 1.90E-03 | 5.32E-02 | NA | NA | NA |  |
| 1 | rs2296172 | 4-(Chloroacetyl)acetanilide | 3.07E-03 | 9.21E-02 | NA | BMP8A, MACF1, OXCT2, OXCT2P1, PABPC4, PPIEL, RP11-69E11.4 | The Neanderthal allele for rs2296172 increases expression of BMP8A in testis and esophagus tissues. BMP proteins have been implicated in cancer progression and suppression and have been proposed as anti-cancer therapeutic targets. | Bach et al., 2017; Pu et al., 2012 |
|  |  | Malachite green oxalate | 1.02E-02 | 2.28E-01 |  |  |  |  |
|  |  | Nifedipine | 5.21E-03 | 1.44E-01 |  |  |  |  |
| 1 | rs2296173 | 4-(Chloroacetyl)acetanilide | 3.29E-03 | 9.21E-02 | NA | BMP8A, MACF1, OXCT2, OXCT2P1, PABPC4, PPIEL, RP11-69E11.4 | The Neanderthal allele for rs2296173 increases expression of BMP8A in testis and esophagus tissues. BMP proteins have been implicated in cancer progression and suppression and have been proposed as anti-cancer therapeutic targets. | Bach et al., 2017; Pu et al., 2012 |
|  |  | Malachite green oxalate | 5.88E-03 | 2.28E-01 |  |  |  |  |
|  |  | Nifedipine | 4.85E-03 | 1.44E-01 |  |  |  |  |
| 1 | rs3765865 | 2,3,5-Trichlorophenol | 4.24E-03 | 2.38E-01 | NA | HHAT, TRAF3IP3, SERTAD4-AS1 | NA |  |
|  |  | Oxymetholone | 3.92E-03 | 9.05E-02 |  |  |  |  |
| 1 | rs4844488 | 2,3,4,5-Tetrachloronitrobenzene | 3.38E-03 | 9.45E-02 | NA | DIEXF | NA |  |
|  |  | Chlorpheniramine maleate | 4.06E-03 | 2.28E-01 |  |  |  |  |
| 2 | rs3771635 | 2-Amino-4-chlorophenol | 1.35E-02 | 1.89E-01 | NA | NA | NA |  |
|  |  | 2,4-Decadienal | 1.88E-03 | 1.05E-01 |  |  |  |  |
|  |  | Diisobutyl phthalate | 4.76E-03 | 1.69E-01 |  |  |  |  |
|  |  | Malachite green oxalate | 1.22E-02 | 2.28E-01 |  |  |  |  |
|  |  | Methacrylonitrile | 1.72E-02 | 2.41E-01 |  |  |  |  |
| 3 | rs12638360 | 5-Fluorouracil | 2.08E-02 | 2.33E-01 | NA | NA | NA |  |
|  |  | t-Butyl formate | 5.00E-03 | 8.03E-02 |  |  |  |  |
| 3 | rs13097326 | 7,12-Dimethylbenzanthracene | 3.32E-03 | 9.31E-02 | NA | CPA3 | NA |  |
| 3 | rs1566479 | Titanocene dichloride | 6.20E-04 | 3.47E-02 | NA | NA | NA |  |
| 3 | rs5186 | 7,12-Dimethylbenzanthracene | 3.03E-03 | 9.31E-02 | NA | CPA3 | NA |  |
|  |  | Nifedipine | 7.73E-03 | 1.44E-01 | NA |  |  |  |
| 3 | rs9855367 | Doxorubicin | 1.79E-03 | 2.28E-01 | NA | NA | NA |  |
| 4 | rs13130029 | Cytarabine | 1.03E-02 | 2.18E-01 | SPATA4, ASB5, MIR1267, WDR17 | ASB5 | The Neanderthal allele for rs13130029 decreases ASB5 expression in heart tissue. ASB5 has been shown to be upregulated in growing collateral arteries and may play a role in the initiation of arteriogenesis. | Boengler et al., 2003 |
|  |  | Sunitinib | 1.17E-02 | 2.50E-01 |  |  |  |  |
| 4 | rs7687260 | Mitoxantrone | 2.98E-04 | 3.78E-02 | NA | NA | NA |  |
| 5 | rs17428724 | Cladribine | 2.72E-03 | 1.22E-01 | LARS, RBM27 | LARS, PLAC8L1, RBM27, SHRF2 | The Neanderthal allele for rs17428724 is associated with increased LARS expression in lung tissue and several other tissue types. LARS expression was shown to be up-regulated in A549 lung cancer cell line and siRNA knockdown experiments suggest possible roles for LARS in the migration and growth of lung cancer cells. | Shin et al., 2008 |
|  |  | Cytarabine | 9.27E-03 | 2.18E-01 |  |  |  |  |
| 5 | rs17618372 | cis-Dichlorodiamine platinum | 3.26E-03 | 1.83E-01 | NA | NA | NA |  |
| 5 | rs2304033 | Cladribine | 4.80E-03 | 1.52E-01 | RBM27 | LARS, PLAC8L1, RBM27, SHRF2 | The Neanderthal allele for rs2304033 is associated with increased LARS expression in lung tissue and several other tissue types. LARS expression was shown to be up-regulated in A549 lung cancer cell line and siRNA knockdown experiments suggest possible roles for LARS in the migration and growth of lung cancer cells. | Shin et al., 2008 |
|  |  | Cytarabine | 7.72E-03 | 2.18E-01 |  |  |  |  |
| 5 | rs3749994 | Cladribine | 2.11E-03 | 1.22E-01 | PLAC8L1, LARS | LARS, PLAC8L1, RBM27, SHRF2 | The Neanderthal allele for rs3749994 is associated with increased LARS expression in lung tissue and several other tissue types. LARS expression was shown to be up-regulated in A549 lung cancer cell line and siRNA knockdown experiments suggest possible roles for LARS in the migration and growth of lung cancer cells. | Shin et al., 2008 |
|  |  |  | 7.73E-03 | 2.18E-01 |  |  |  |  |
| 5 | rs7443214 | Cladribine | 2.88E-03 | 1.22E-01 | LARS, RBM27 | LARS, PLAC8L1, RBM27, SHRF2 | The Neanderthal allele for rs7443214 is associated with increased LARS expression in lung tissue and several other tissue types. LARS expression was shown to be up-regulated in A549 lung cancer cell line and siRNA knockdown experiments suggest possible roles for LARS in the migration and growth of lung cancer cells. | Shin et al., 2008 |
| 6 | rs10455593 | Sunitinib | 1.18E-02 | 2.50E-01 | NA | NA | NA |  |
| 6 | rs10457421 | t-Butyl formate | 7.98E-03 | 8.93E-02 | NA | GJA1 | The Neanderthal allele for rs10457421 increases GJA1 expression in sun-exposed skin tissue. GJA1 (connexin43) expression has been shown to be downregulated in carcinoma cells and its role in cancer may be tissue-and stage-specific. | Busby et al., 2018; King et al., 2000; Yi et al., 2007 |
| 6 | rs12201990 | Ninhydrin | 2.53E-03 | 7.08E-02 | NA | GJA1 | The Neanderthal allele for rs12201990 increases GJA1 expression in sun-exposed skin tissue. GJA1 (connexin43) expression has been shown to be downregulated in carcinoma cells and its role in cancer may be tissue-and stage-specific. | Busby et al., 2018; King et al., 2000; Yi et al., 2007 |
|  |  | p-tert-Butylcatechol | 1.30E-02 | 2.43E-01 |  |  |  |  |
|  |  | t-Butyl formate | 3.03E-05 | 1.69E-03 |  |  |  |  |
| 6 | rs12209650 | Sunitinib | 6.51E-03 | 2.50E-01 | HTR1B | NA | Silencing of the HTR1B gene via hypermethylation of an upstream CpG island was observed in human squamous cell lung cancer cell lines. | Takai et al., 2001 |
| 6 | rs1563929 | Sunitinib | 6.90E-03 | 2.50E-01 | NA | NA | NA |  |
| 6 | rs4714738 | N,N'-Di-sec-butyl-p-phenyldiamine | 8.46E-04 | 4.74E-02 | NA | TMEM63B | The Neanderthal allele for rs4714738 increases TMEM63B expression in multiple tissue types. TMEM63B expression has been shown to be up-regulated in hepatocellular carcinoma tissue samples. | Chiang et al., 2008 |
| 6 | rs9369453 | 5-Fluorouracil | 9.76E-03 | 1.82E-01 | NA | TMEM63B, MRPL14 | The Neanderthal allele for rs9369453 increases TMEM63B expression in multiple tissue types. TMEM63B expression has been shown to be up-regulated in hepatocellular carcinoma tissue samples. | Chiang et al., 2008 |
| 8 | rs17079571 | Diglycidyl resorcinol ether (DGRE) | 4.33E-03 | 2.42E-01 | NA | NA | NA |  |
| 9 | rs10512230 | 2-Biphenylamine | 8.07E-03 | 2.40E-01 | PTPDC1, MIRLET7A1, MIRLET7F1, MIRLET7D | RP11-307E17.8, ZNF169, HIATL1 | The Neanderthal allele for rs10512230 increases ZNF169 and RP11-307E17.8 expression and decreases HIATL1 expression. The genomic region between ZNF16 and FANCC has been shown to be associated with multiple self-healing epitheliomata. The LET-7 family of microRNAs regulate RAS expression in human cells, which is upregulated in lung tumors, thus providing a possible role for LET-7 microRNAs in human lung carcinogenesis. | Bose et al., 2006; Johnson et al., 2005; Yanaihara et al., 2006 |
|  |  | Chlordane (technical grade) | 3.75E-03 | 2.07E-01 |  |  |  |  |
|  |  | Cytarabine | 1.22E-02 | 2.21E-01 |  |  |  |  |
|  |  | Nitazoxanide | 2.58E-03 | 1.45E-01 |  |  |  |  |
|  |  | tetra-N-Octylammonium bromide | 2.23E-03 | 1.25E-01 |  |  |  |  |
| 9 | rs10978949 | Sunitinib | 5.93E-03 | 2.50E-01 | NA | NA | NA |  |
| 9 | rs10981252 | 2-Amino-4-chlorophenol | 7.68E-03 | 1.89E-01 | NA | NA | NA |  |
| 9 | rs17355458 | Cytarabine | 6.27E-04 | 3.98E-02 | NA | NA | NA |  |
| 9 | rs7039165 | Oxymetholone | 3.65E-03 | 9.05E-02 | NA | LPAR1 | The Neanderthal allele for rs7039165 decreases LPAR1 expression in lung tissue. LPAR1 has been implicated in cancer progression and invasion in prostate cancer cells. Mutations in LPAR1 were identified via transcriptome sequencing in patients with metastatic neuroblastoma. | Ward et al., 2011; Wei et al., 2013 |
| 9 | rs7850580 | Cytarabine | 3.57E-04 | 3.98E-02 | NA | NA | NA |  |
| 10 | rs11248442 | o-Phenylenediamine | 3.66E-03 | 2.05E-01 | NA | NA | NA |  |
| 10 | rs2453 | 2-Amino-4-chlorophenol | 1.34E-02 | 1.89E-01 | NA | NA | NA |  |
|  |  | Methacrylonitrile | 9.12E-03 | 1.99E-01 |  |  |  |  |
|  |  | tetra-N-Octylammonium bromide | 7.75E-03 | 1.63E-01 |  |  |  |  |
|  |  | Ziram | 8.50E-03 | 1.81E-01 |  |  |  |  |
| 11 | rs10892201 | Hydroquinone | 5.26E-04 | 2.95E-02 | NA | NA | NA |  |
| 11 | rs12790857 | Temozolomide | 1.41E-03 | 1.14E-01 | TSPAN18 | NA | TSPAN18 is a member of the tetraspanins family of proteins which are known to play important roles in cellular adhesion, tumor growth, invasion and metastasis. | Zijlstra, 2009; Sala-Valdes et al., 2012 |
| 11 | rs2156528 | 4,4-Thiobis(6-tert-butyl-m-cresol) | 9.65E-04 | 5.40E-02 | NA | MMP7 | The Neanderthal allele for rs2156528 increases MMP7 expression in sun-exposed skin tissue. MMP-7 expression was found to be correlated with metastases and invasion in human colorectal cancers, ovarian cancer, and gastrointestinal carcinomas. | Shiomi et al., 2003; Adachi et al., 1999; Wang et al., 2004 |
|  |  | trans-1,4-dichloro-2-butene | 2.30E-03 | 1.29E-01 |  |  |  |  |
| 11 | rs2307073 | Hematoxylin | 8.26E-03 | 2.31E-01 | NA | USP47 | The Neanderthal allele for rs2307073 decreases USP47 expression in whole blood and esophagus (mucosa) tissue. Inhibition of USP47 has been shown to inhibit proliferation in gastric cancer cells, multiple myeloma and other cancers. | Weinstock et al., 2012; Zhang et al., 2014 |
|  |  | Iodochlorohydroxyquinoline | 1.84E-03 | 1.03E-01 |  |  |  |  |
|  |  | t-Butyl formate | 5.74E-03 | 8.03E-02 |  |  |  |  |
| 11 | rs7110434 | Temozolomide | 1.80E-03 | 1.14E-01 | TSPAN18 | NA | TSPAN18 is a member of the tetraspanins family of proteins which are known to play important roles in cellular adhesion, tumor growth, invasion and metastasis. | Zijlstra, 2009; Sala-Valdes et al., 2012 |
| 12 | rs11049548 | 5-Fluorouracil | 6.89E-03 | 1.82E-01 | NA | CCDC91, RP11-967K21.1, | The Neanderthal allele for rs11049548 increases CCDC91 expression in multiple tissue types. CCDC91 has been shown to be associated with the development of breast cancer. | Freeman et al., 2015 |
| 12 | rs12423929 | Pyrithione zirconium | 2.83E-03 | 1.58E-01 | NA | RP11-341G23.4 | NA |  |
| 12 | rs12427160 | Chlordane (technical grade) | 7.38E-03 | 2.07E-01 | NA | NA | NA |  |
|  |  | Ninhydrin | 2.18E-03 | 7.08E-02 |  |  |  |  |
| 12 | rs12811504 | 2-Amino-4-chlorophenol | 9.49E-03 | 1.89E-01 | NA | NA | NA |  |
|  |  | 3,4-Dinitrotoluene | 1.48E-03 | 8.26E-02 |  |  |  |  |
|  |  | 6-Thioguanine (6-TG) | 1.20E-03 | 6.74E-02 |  |  |  |  |
|  |  | Diisobutyl phthalate | 6.05E-03 | 1.69E-01 |  |  |  |  |
|  |  | Hematoxylin | 6.65E-03 | 2.31E-01 |  |  |  |  |
|  |  | Oxymetholone | 4.85E-03 | 9.05E-02 |  |  |  |  |
|  |  | t-Butyl formate | 2.50E-03 | 6.99E-02 |  |  |  |  |
|  |  | Ziram | 5.04E-03 | 1.81E-01 |  |  |  |  |
| 12 | rs2200275 | 2-Biphenylamine | 8.58E-03 | 2.40E-01 | NA | NA | NA |  |
|  |  | Chlordecone (kepone) | 7.98E-03 | 2.23E-01 |  |  |  |  |
|  |  | p-tert-Butylcatechol | 1.11E-03 | 6.23E-02 |  |  |  |  |
| 12 | rs4761177 | Mitoxantrone | 3.17E-03 | 1.34E-01 | NA | CNOT2, KCNMB4, RP11-320P7.1, RP11-611E13.2, | The Neanderthal allele for rs4761177 decreases CNOT2 expression in subcutaneous adipose tissue. Down-regulation of CNOT2 using siRNA was shown to induce apoptosis by inhibiting the CCR4-NOT deadenylase complex. | Ito et al., 2011; White et al., 2012 |
| 12 | rs7979823 | Mitoxantrone | 3.17E-03 | 1.34E-01 | NA | CNOT2, KCNMB4, RP11-320P7.1, RP11-611E13.2, | The Neanderthal allele for rs7979823 decreases CNOT2 expression in subcutaneous adipose tissue. Down-regulation of CNOT2 using siRNA was shown to induce apoptosis by inhibiting the CCR4-NOT deadenylase complex. | Ito et al., 2011; White et al., 2012 |
| 13 | rs16971270 | Chlordecone (kepone) | 5.14E-04 | 2.88E-02 | NA | NA | NA |  |
|  |  | p-Benzoquinone dioxime | 3.42E-03 | 1.91E-01 |  |  |  |  |
| 13 | rs9316483 | 1,8-Dihydroxy-4,5-dinitroanthraquinone | 1.86E-03 | 1.04E-01 | NA | MICU2, ZDHHC20 | ZDHHC20 is a protein in the palmitoyl acyltransferase (PAT) enzymes family, which are targets for anticancer compounds. Overexpression of ZDHHC20 was shown to increase cell proliferation in NIH/3t3 cells. | Draper et al., 2011; Anderson et al., 2016; Ducker et al., 2006 |
| 14 | rs2400963 | 2,3,4,5-Tetrachloronitrobenzene | 1.14E-03 | 6.38E-02 | SNORD-112, SNORD113-1to9, SNORD114-1to19, RTL1, MEG8, MIR431, MIR433, MIR127, MIR432, MIR136 | AL132709.5, RP11-909M7.3 | The Neanderthal allele of rs2400963 increases expression of AL132709.5 in testis tissue and decreases expression of RP11-909M7.3 in transformed fibroblasts. These miRNAs are located in the DLK-DIO3 locus. Several miRNAs in the DLK-DIO3 locus have been implicated in various cancer pathways. | Dai et al., 2016; Benetatos et al., 2013; Valleron et al., 2012; Luk et al., 2011; Xu et al., 2014 |
|  |  | Amiloride hydrochloride | 3.97E-04 | 2.22E-02 |  |  |  |  |
|  |  | Nilotinib | 1.05E-02 | 1.99E-01 |  |  |  |  |
| 14 | rs2886122 | 5-Fluorouracil | 1.58E-02 | 2.22E-01 | NA | SPTLC2, ADCK1, SNW1, SLIRP, GSTZ1 | The Neanderthal allele for rs2886122 increases SPTLC2 expression in tibial nerve tissue. SPTLC2 activity has been shown to cause ceramide-dependent cell death. Inhibition of SPTLC2 via phosphorylation, inhibits its activity and prevents ceramide-dependent cell death. | Taouji et al., 2013; Bieberich, 2008 |
|  |  | Methacrylonitrile | 1.06E-02 | 1.99E-01 |  |  |  |  |
|  |  | Methylene bis(thiocyanate) | 2.17E-04 | 1.22E-02 |  |  |  |  |
|  |  | Tetraethylene glycol diacrylate | 2.35E-03 | 1.32E-01 |  |  |  |  |
|  |  | Ziram | 1.59E-02 | 2.22E-01 |  |  |  |  |
| 16 | rs10514412 | t-Butyl formate | 1.63E-02 | 1.52E-01 | NA | ADAMTS18 | The Neanderthal allele for rs10514412 increases ADAMTS18 expression in brain tissue. Epigenetic inactivation of ADAMTS18 was identified to act as a tumor suppressor in multiple carcinomas. Mutations in the ADAMTS18 were identified to promote growth, migration and metastasis in melanoma. | Jin et al., 2007; Wei et al., 2010 |
| 16 | rs4784260 | ArsenicTrioxide | 4.97E-04 | 6.31E-02 | NA | NA | NA |  |
|  |  | Azacitidine | 1.55E-03 | 1.97E-01 |  |  |  |  |
|  |  | Sunitinib | 1.06E-02 | 2.50E-01 |  |  |  |  |
| 16 | rs4887941 | Dichlorvos (Vapona) | 3.02E-03 | 1.69E-01 | NA | CLEC3A | The Neanderthal allele for rs4887941 increases CLEC3A expression in testis tissue. CLEC3A promotes cell adhesion by binding to heparan sulfate proteoglycans on the cell surface. Cleavage of CLEC3A by MMP-7 diminishes adhesion and promotes migration. | Tsunezumi et al., 2009 |
| 17 | rs4968063 | N-(1-Naphthyl) ethylenediamine dihydrochloride | 3.22E-03 | 1.80E-01 | NA | NA | NA |  |
|  |  | tetra-N-Octylammonium bromide | 1.25E-02 | 1.63E-01 |  |  |  |  |
